# Bleaching gel volume influences hydrogen peroxide diffusion, inflammation, and the presence of nitric oxide in the pulp tissue: *in vitro* and *in vivo* model

**DOI:** 10.1590/1678-7757-2023-0265

**Published:** 2023-12-18

**Authors:** Sibele de ALCÂNTARA, Francine BENETTI, Lívia Maria Alves Valentim da SILVA, Nathália Evelyn da Silva MACHADO, Isabela Joane Prado SILVA, Lara Maria Bueno ESTEVES, Edilson ERVOLINO, Luciano Tavares Angelo CINTRA, André Luiz Fraga BRISO

**Affiliations:** 1 Universidade Estadual Paulista Faculdade de Odontologia de Araçatuba Departamento de Odontologia Preventiva e Restauradora Araçatuba SP Brasil Universidade Estadual Paulista (UNESP), Faculdade de Odontologia de Araçatuba, Departamento de Odontologia Preventiva e Restauradora, Araçatuba, SP, Brasil.; 2 Universidade Federal de Minas Gerais Faculdade de Odontologia Departamento de Odontologia Restauradora Belo Horizonte MG Brasil Universidade Federal de Minas Gerais, Faculdade de Odontologia, Departamento de Odontologia Restauradora, Belo Horizonte, MG, Brasil.; 3 Universidade Estadual Paulista Faculdade de Odontologia de Araçatuba Departamento de Ciências Básicas Araçatuba SP Brasil Universidade Estadual Paulista (UNESP), Faculdade de Odontologia de Araçatuba, Departamento de Ciências Básicas, Araçatuba, SP, Brasil.

**Keywords:** Tooth bleaching, Oxidative stress, Nitric oxide, Dosage, Hydrogen peroxide

## Abstract

**Objective:**

To assess whether bleaching gel volume influences chromatic changes, hydrogen peroxide (HP) diffusion, inflammation, and oxidative stress in the pulp tissue.

**Methodology:**

A total of 60 bovine teeth were divided into four groups, according to bleaching gel volume (n=15): without gel (WG); V30 (30 µL of 35% HP); V60 (60 µL); and V120 (120 μL). HP diffusion analysis was performed in the first session (T1). Chromatic changes (ΔE, ΔE00, and WID) were assessed after the first (T1), second (T2), third (T3) sessions, and 15 d (T4) after the end of treatment. Moreover, 20 rats were randomly divided into four groups (n=10) and their upper first molars were treated with different gel volumes: control (no treatment); V2 (2 μL of 17.5% HP); V4 (4 μL); and V8 (8 μL). After 24 h, rats were euthanized and the specimens processed for histological and immunohistochemical (nitric oxide synthase) evaluation. Data were analyzed using the Wilcoxon and Mann-Whitney tests (p<0.05).

**Results:**

*In vitro* (bovine teeth), chromatic changes were not influenced by bleaching gel volume, showing similar values in all groups and sessions, except for the control group (p<0.05). The V120 group had the highest HP diffusion values (p<0.05). *In vivo* (pulp tissue), the V4 and V8 groups showed the highest inflammatory infiltrate in the pulp and significant oxidative stress (p<0.05).

**Conclusion:**

The adverse effects on the dental pulp related to HP diffusion, pulp inflammation, and oxidative stress depend on bleaching gel volume, while the bleaching effect is not proportional to the volume used.

## Introduction

Several techniques and products have been developed to improve tooth bleaching results, mainly by using highly concentrated products, as indicated for in-office procedures.^1,2^ However, these techniques can lead to postoperative tooth sensitivity^3^ and changes in the pulp, such as severe inflammation and tissue necrosis, as assessed in human teeth^4^ and animal studies.^5-7^ This is due to the low molecular weight of the reactive oxygen species (ROS) of hydrogen peroxide (HP) in bleaching gels, which diffuse into the enamel and dentin, oxidizing chromophore agents, thus resulting in teeth with lighter shades.^8^

Oxidative stress in the pulp tissue can enhance the production and release of biochemical agents^9,10^ such as nitric oxide (NO). NO is produced endogenously by nitric oxide synthase (NOS) and, when generated by the inducible form of NOS (iNOS), is crucial for nonspecific host defense and innate immunity.^11^ However, this enzyme can trigger the sustained release of reactive species that damage various biomolecules, resulting in NO-associated inflammation.^11^ Despite its importance as a marker of tissue oxidative stress, iNOS has not yet been evaluated in the pulp tissue of bleached teeth. Considering the plurality of effects of NO on the dental pulp, assessing its participation in events that potentially induce pain and inflammation is important.

Previous studies showed that longer exposure times and bleaching gel concentration intensify pulp damage.^12^ A clinical study^13^ observed that the amount of whitening gel used during therapy can be decisive for both color change response and postoperative sensitivity. However, the literature still lacks answers on the influence of bleaching gel volume on pulp tissue damage. Therefore, understanding the effect of bleaching gel volume is necessary in order to assess whether maintaining the effect using milder concentrations could represent a considerable advantage in the biological response to this procedure.

Thus, this study sought to evaluate the influence of bleaching gel volume on chromatic changes, HP diffusion, the inflammatory process, and oxidative stress in the dental pulp, considering as a null hypothesis that the bleaching gel volume used does not influence: (1) chromatic changes; (2) HP diffusion; (3) inflammatory response; and (4) oxidative stress in the pulp tissue.

## Methodology

### Experimental design of the *in vitro* study

The sample size was calculated based on data from a previous study^10,14^ (difference in means of 2.8, standard deviation of 2.2, with a significance level α of 0.05 and a power test 1-β of 0.80), using SigmaPlot 12.0 (Systat, San Jose, CA, USA). It consisted of 60 tooth discs (n=15), initially obtained from 120 permanent bovine incisors, excluding teeth with morphological changes in the crown and enamel cracks, stains, or excessive incisor wear. The teeth were stored in physiological saline solution with 0.1% thymol under refrigeration at approximately 4°C and remained so until the beginning of the experimental phase.^2^ The study was approved by the university’s Research Ethics Committee (Process 547/2019).

Subsequently, the roots were separated from the crowns at the cementoenamel junction, using an 8 mm diameter diamond glass cutting tip (Dinser Diamond Tools Ltda., Sacomã, SP, Brazil), under constant irrigation, obtaining enamel/dentin discs with a diameter of 5.7 mm.^2^ The dentin surface was smoothed by manual rotational movements on 600-grit aluminum oxide sandpaper (T469-SF-Noton, Saint-Gobain Abrasives Ltda., Jundiaí, SP, Brazil) until it reached a thickness of 3.7 mm (1.3 mm of enamel and 2.4 mm of dentin; ±0.2 mm), measured with a digital caliper (500-144B, Mitutoyo Sul América Ltda., SP, Brazil). The smear layer was removed by applying 0.5 M EDTA solution, pH 7.2, for 30 seconds on the dentin surface, followed by rinsing with deionized water.^2^

The E* reflectance value dials were then selected with the visible E* value obtained by UV-2450 spectrophotometer (Shimadzu, Kyoto, Japan), adjusted to the CIE L*a*b* color scale, with D65 illuminant, fast scanning speed, and 380 to 780 nm measurement mode, which uses the CIELAB* color space, also referred to as L*a*b*. The L* axis (L*=100) refers to the resolution between white (L*=100) and black (L*=0); the a* axis is relative to the green-red opponent colors, ranging from −90 to +70, with negative values for greenish colors and positive values for reddish colors; and the b* axis represents the blue-yellow scale, ranging from −80 to +100, with negative values for bluish colors and positive values for yellowish colors.^14^ The values were calculated using the formula $E={\left[(L{)}^{2}+(a{)}^{2}+(b{)}^{2}\right]}^{1/2}$ . After obtaining these, the 60 discs with the lowest mean values were selected, with values of 66.73 and a standard of 0.10.

The selected samples were separated into four experimental groups according to gel volume (n=15): without gel (WG); V30 (30 µL of 35% HP); V60 (60 µL of 35% HP); and V120 (120 µL of 35% HP). The gel volume used was determined using the only information provided by the manufacturer in the product leaflet, which suggests that one tube would be enough for four sessions on human teeth, whitening 20 teeth per session. Thus, in the *in vitro* study, when the gel was applied to the study samples, maintaining an average thickness of 1 mm, a volume of 60 µl was found, considered the base value. Once this value was obtained, the analysis was performed with half (30 µL) and double (120 µL), thus determining the three volumes tested in the study. The sample was then placed in an artificial pulp chamber (APCs), adapted to its internal compartment and sealed between two rubbers that were kept with the fittings adjusted to the APC with an accuracy of 1 mm.

### Bleaching procedure and color analysis

Bleaching was performed using Whiteness HP Maxx (FGM Produtos Odontológicos, Joinville, SC, Brazil) based on 35% HP, manipulated in a 3:1 ratio, according to the manufacturer’s instructions^9^. Gel volumes of 30 µL, 60 µL, and 120 µL were applied for 45 minutes to the enamel of the samples from the respective experimental groups using a pipette (Microman E M100E, Gilson, Inc., Wisconsin, USA).^10^ The gel was then removed using suction cups and fixed papers and washed in running water. The procedure was repeated two more times with an interval of one week between sessions, storing the samples in humidifying chambers between sessions until the end of the whole experiment and the treatment performed in a single application.

Chromatic changes (∆E and ∆E_00_) and the whitening index (W_ID_) were assessed 24 h after the end of each session, classified as T1 (after the first bleaching session), T2 (after the second bleaching session), T3 (after the third bleaching session), and T4 (15 d after the end of treatment), using an ultraviolet-visible spectrophotometer, both based on analysis of the CIELAB* axes system. ∆E and ∆E_00_, were calculated using the formulas: $\Delta E={\left[(\Delta L{)}^{2}+(\Delta b{)}^{2}\right]}^{1/2}$ . The whitening index was obtained using the following formula: $\Delta E_{00=}[{\left(\Delta L^{\prime }/KLSL\right)}^{2}+(\Delta C^{\prime }/KCSC{)}^{2}+{\left(\Delta H^{\prime }/KHSH\right)}^{2}+{RT}^{*}\left(\Delta C^{\prime }/KCSC\right)*(\Delta H^{\prime }/KHSH){]}^{1/2}$ .^15^

### Transenamel and Transdentinal HP diffusion

**Figure 1:**
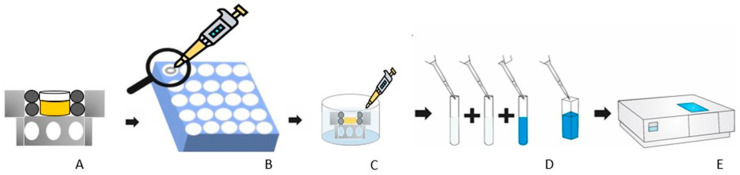
Transenamel and Transdentinal HP diffusion protocol

Transenamel and Transdentinal HP diffusion was analyzed only in the first bleaching session. Each enamel/dentin disc was individually adapted to an APC. Each APC had two compartments: at the top, an opening with a diameter of 8 mm and, in the second compartment, an opening with a diameter of 6 mm allowed the sample to be properly positioned and sealed from the side. The lower portion had lateral perforations for the circulation of the solution and was used to quantify the peroxide penetrating the specimen (Figure 1).

For this purpose, the sample was positioned in an APC, with its compartment adapted as the upper one, between two that were kept with the fittings adjusted to the APCs,^16^ with the addition of 1 mL of acetate buffer, made from 2 M acetate and 2 M acetic acid solutions with pH 4.^16^ The solution in the APC filler remains in contact with the dentin surface, while the white gel remains on the enamel, allowing it to stabilize in solution in a lower environment.^8^

After removing the solution and the gel, leucocrystal violet (0.5 mg/mL; Sigma Chemical Co, St Louis, MO, USA) and peroxidase enzyme (1 mg/mL; Sigma Chemical Co, St Louis, MO, USA) were added to the solution. This method analyzes the reaction between HP and leucocrystal violet dye, catalyzed by the enzyme peroxidase.^16^ The resulting mixture varies according to the amount of peroxide diffused into the solution.

Reflectance measurements of the standard and spectral solutions in APCs were performed without visible reflectance (5UV-20, Shimadzu), at a wavelength of 596 nm. The data generated an absorption × concentration graph, which determined HP diffusion into the tooth structure and stabilization within the APC.

### Experimental design of the *in vivo* study

The *in vivo* study was conducted to assess inflammation and the presence of nitric oxide in the pulp. A total of 20 8-week-old male Wistar rats (*Rattus Albinus*), weighing approximately 250 g, were used. The sample size was calculated using data from previous studies.^17^ Rats were kept in an environment with a temperature of 22°C to 24°C, in a controlled light cycle (12 h light and 12 h dark), with food and water *ad libitum* (Mogiana Alimentos SA, Campinas, Brazil). The study was approved by the university’s Research Ethics Committee (Process 547/20) and by the NIH Guide for the Care and Use of Laboratory Animals (Bethe, MD, USA).

### Bleaching treatment

Before the bleaching procedure, rats received an intramuscular injection of the combined sedative xylazine hydrochloride 2% (10 mg/kg) and ketamine hydrochloride 10% (80 mg/kg). The 17.5% HP-based bleaching gel was obtained by diluting the 35% HP-based gel (Whiteness HP Maxx, FGM Produtos Odontológicos, Joinville, SC, Brazil), which is marketed in two bottles, one with HP and the other with thickener. The 17.5% HP gel was used in a 3:3:2 ratio, with three drops of HP, three drops of distilled water, and two drops of thickener^9^. After application and photoactivation of the resin gingival barrier (Top Dam, FGM Produtos Odontológicos, Joinville, SC, Brazil), the right or left upper molars of rats were randomly divided into four groups: control ( – the molars were not treated; V2 – 2 µL of 17.5% HP gel was applied to the molars with a pipette for viscous liquids (Microman E M100E, Gilson, Inc., Middleton, Wisconsin, USA) and left in contact with the tooth for 10 minutes; the teeth were then cleaned with cotton and rinsed thoroughly; V4 – 4 µL of 17.5% HP gel was applied for 10 minutes; and V8 – 8 µL of 17.5% HP gel was applied for 10 minutes. The exposure time and concentration were determined by published studies^18^ and pilot tests, which showed that 4 µL of gel produced inflammation but not necrosis of the pulp tissue.

### Laboratory steps

Rats were euthanized 24 hours after the bleaching session by an anesthetic overdose of sodium thiopental (150 mg/kg; Thipentax, Cristália Produtos Farmacêuticos Ltda., Itapira, Brazil). The hemimaxillae were separated, dissected, and fixed in a 4% buffered formaldehyde solution for 24 h. The specimens were then decalcified in a 10% ethylenediaminetetraacetic acid (EDTA) solution (pH 7) for approximately three months, dehydrated, clarified, and embedded in paraffin.

The sections were selected when the mesial root of the first molar was visible in its entire longitudinal extent.^7,19^ Subsequently, 5 µm sections were cut in the bucco-lingual plane using a microtome (RM 2045, Leica Microsystems, Wetzlar, Germany). Slides with histological sections were then stained with hematoxylin-eosin or obtained for immunohistochemical evaluations using an indirect immunoperoxidase technique.^7,19^

For the immunohistochemical reaction,^7,19^ histological sections were deparaffinized in xylene and hydrated in a decreasing series of ethanol (100–100–100–90–70 GL). For antigen retrieval, the slides were immersed in citrate buffer, pH 6, in a pressurized chamber (Decloaking Chamber, Biocare Medical, Concord, CA, USA) for 10 minutes at 95°C. After washing in 0.1 M, pH 7.4, the histological slides were immersed in 3% HP for 1 h to block endogenous peroxidase. After washing in phosphate buffered saline (PBS), the histological sections were treated with 3% serum albumin for 12 h to block nonspecific sites. The histological slides were then incubated with the primary anti-NOS antibody (rabbit-generated mouse antibody, LS-B3657-250, LifeSpan BioSciences, WA, USA). This primary antibody was diluted in PBS plus 0.1% Triton X-100 (PBS-TX) for 24 h in a moist chamber. After washing, the sections were incubated with the biotinylated secondary antibody for 2 h, washed again, and treated with horseradish peroxidase-conjugated streptavidin for 1 h (Universal Dako Labeled HRP Streptavidin-Biotin Kit^®^, Dako, CA, USA). Three washes were then performed in PBS-TX and the reaction was produced using 3,3’-diaminobenzidine tetrahydrochloride (DAB Chromogen Kit^®^, Dako, CA, USA) as a chromogen. After a series of washes in PBS, the histological sections were counterstained with Harris hematoxylin. As a negative control, the new specimens underwent the same procedures, except for the use of the primary antibody.

### Histological and immunohistochemical analysis

A single blinded and calibrated operator performed histological and immunohistochemical analyses using an optical microscope (DM 400 B, Lei^®^, Wetzlar, Germany). For the analysis of the inflammatory infiltrate, scores were assigned to each third of the pulp chamber (occlusal, middle, and cervical):^7,19^ 1 – absence or insignificant number of inflammatory cells; 2 – mild inflammatory infiltrate (less than 25 cells per field); 3 – moderate inflammatory infiltrate (25 to 125 cells per field); 4 – severe inflammatory infiltrate (more than 125 cells per field); and 5 – necrosis.

iNOS immunolabeling was analyzed by assessing the presence of a brownish stain in the cytoplasm of the cells and in the extracellular matrix, considering the following score: 0 – no immunolabeling (no labeling in the extracellular matrix and complete absence of immunoreactive cells); 1 – low immunolabeling pattern (weak labeling in the extracellular matrix and approximately a quarter of immunoreactive cells); 2 – moderate immunolabeling pattern (moderate labeling in the extracellular matrix and approximately half of immunoreactive cells); 3 – severe immunolabeling pattern (strong labeling in the extracellular matrix and approximately three quarters of immunoreactive cells); and 4 – very severe immunolabeling pattern (extremely strong labeling in the extracellular matrix and approximately all immunoreactive cells).^7,19^

### Statistical analysis

Statistical analysis was performed using SigmaPlot (SYSTAT, San Jose, CA, USA). After the normality test, data on chromatic changes (∆E) and the whitening index (∆W_ID_) were subjected to two-way repeated measures ANOVA, and HP diffusion data were analyzed by one-way ANOVA, followed by Tukey’s test (p<0.05).

Nonparametric data (inflammatory response and immunolabeling) were analyzed by the Kruskal-Wallis test followed by Dunn’s test (*P*<0.05).

## Results

### Chromatic change analysis

Table 1 presents the results for ΔE, ΔE_00_, and ΔW_ID_. Regarding ΔE, chromatic changes were independent of the gel volume used, with WG showing lower values than the other groups analyzed (p<0.05). The behavior of the groups over time showed that WG and V120 kept their values unchanged from the first bleaching session (T1) until the end of treatment. On the other hand, V30 and V60 showed the highest values only after the second bleaching session.

**Table 1:** Mean (±standard deviation)* of ΔE, ΔE00, and ΔWID analyses

Analysis	Groups	T1	T2	T3	T4
ΔE	WG	2.46 (±1.69)^Ba^	2.34 (±2.21)^Ba^	3.16 (±2.60)^Ba^	3.28 (±2.24)^Ba^
	V30	5.35 (±1.60)^Ab^	6.67 (±1.99)^Aa^	5.72 (±2.56)^Aab^	6.60 (±1.90)^Aa^
	V60	5.00 (±1.50)^Ab^	6.06 (±2.02)^Aab^	5.90 (±2.68)^Aab^	6.86 (±2.56)^Aa^
	V120	5.85 (±1.48)^Aa^	6.09 (±2.27)^Aa^	6.16 (±2.75)^Aa^	6.87 (±2.53)^Aa^
ΔE00	WG	3.57 (±2.31)^Ba^	3.61 (± 2.61)^Ba^	3.78 (±1.90)^Ba^	3.76 (±2.13)^Ba^
	V30	5.98 (±2.51)A^Bb^	6.68 (± 1.93)^ABab^	5.71 (±2.07)^ABb^	7.11 (±1.97)^Aa^
	V60	6.22 (±2.22)^Aa^	7.17 (± 2.44)^Aa^	7.43 (±2.18)^Aa^	6.70 (±2.30)^Aa^
	V120	5.67 (±2.29)^Ab^	7.37 (± 2.75)^Aab^	6.02 (±2.50)^Ab^	7.91 (±3.18)^Aa^
ΔWID	WG	−1.31 (±1.68)^Bab^	−0.45 (±1.78)^Ba^	−1.81 (±1.18)^Bab^	−2.15 (±2.46)^Bb^
	V30	3.92 (±3.13)^Ab^	5.50 (±1.93)^Aa^	3.55 (±3.90)^Ab^	4.73 (±3.23)^Aab^
	V60	3.83 (±3.54)^Aa^	3.39 (±3.39)^Aa^	3.56 (±3.69)^Aa^	4.23 (±3.99)^Aa^
	V120	5.51 (±3.23)^Aab^	4.24 (±3.77)^Ab^	4.70 (±3.61)^Aab^	6.07 (±3.91)^Aa^

*Different uppercase and lowercase letters indicate a significant difference between the groups in each period evaluated and within each group in the different periods evaluated, respectively (p<0.05).

The ΔE_00_ analysis showed similar results for the V30, V60, and V120 groups in all periods. Moreover, V30 was similar to WG in T1, T2, and T3. When analyzing the behavior of each group individually, we observed that WG and V60 remained unchanged over time, while V30 and V120 had the highest ΔE_00_ values at T2.

Similarly, the ΔW_ID_ analysis showed that the gel volume did not influence the bleaching effect, which was equally effective in all groups, except for the control group (p<0.001). When analyzing the behavior of the groups over time, we noted that, despite temporary oscillations, the results obtained after the first bleaching session (T1) were similar to those observed at the end of the experiment (T4) in all groups.

### HP diffusion analysis

Table 2 shows the mean HP concentration that permeated the tooth tissue and reached the APC. The WG group had the lowest significant values, differing from the other groups (p<0.001). The HP penetration values also differed significantly between the experimental groups (p<0.001), with the V120 group showing the highest values, followed by the V60 and V30 groups.

**Table 2:** Mean (±standard deviation) of HP concentration (μg/mL) permeated

Groups	HP concentration*
WG	0.101 (0.10)^a^
V30	0.849 (0.35)^b^
V60	1.142 (0.42)^c^
V120	1.782 (0.30)^d^

*Different letters indicate a statistically significant difference between the groups (p<0.05).

### Inflammatory infiltrate analysis

**Figure 2:**
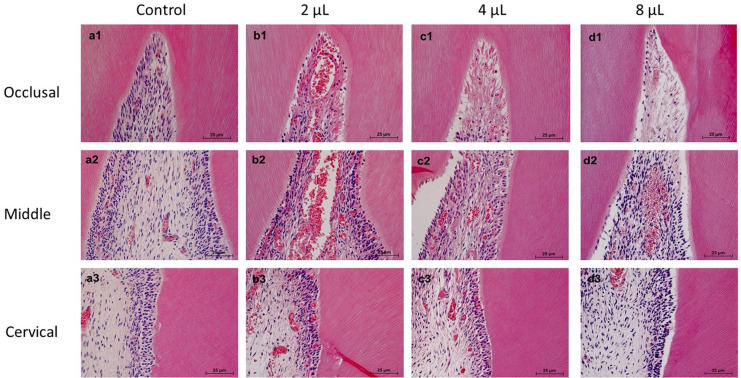
Representative images of the inflammatory infiltrate. (A) Control group: Absence of inflammatory cells in the occlusal (a1), middle (a2), and cervical (a3) thirds. (B) V2: Areas of moderate (b1), mild (b2), and absent (b3) inflammatory infiltrate. (C) V4: Severe (c1), mild (c2), and absent (c3) inflammatory infiltrate. (D) V8: Areas of necrosis (d1) and moderate (d2) and absent (d3) inflammatory infiltrate. [Hematoxylin-eosin staining: 100x and 400x]

Figure 2 shows representative images of the analysis of the inflammatory infiltrate, and the results are described in Table 3. The WG group had no inflammation in any third of the dental pulp, while the V2 group showed less inflammatory infiltrate in the occlusal third than the V4 and V8 groups (p<0.05). V4 and V8 were significantly similar, with necrosis observed in the V8 group and severe inflammatory infiltrate in the V4 group (p>0.05).

**Table 3:** Score assigned to the inflammatory infiltrate and immunolabeling in each third of the pulp chamber

Analysis	Groups	Occlusal	Middle	Cervical
H&E	WG	1^A^	1^A^	1^A^
	V2	3^AB^	2^AB^	1^A^
	V4	4^B^	2^B^	1^A^
	V8	5^B^	2^B^	1^A^
NOS immunolabeling	WG	1^A^	1^A^	1^A^
	V2	2^AB^	2^AB^	1^A^
	V4	3^B^	2^A^	1^A^
	V8	3^B^	3^B^	2^A^

*Different letters in the lines indicate a statistically significant difference between the groups in each analysis (p<0.05).

In the middle third, all the bleached groups showed a mild inflammatory infiltrate, although the V2 and WG groups had similar results (p>0.05). In the cervical third, all groups showed organized pulp tissue and no inflammatory cells (p>0.05).

### NOS immunolabeling

**Figure 3:**
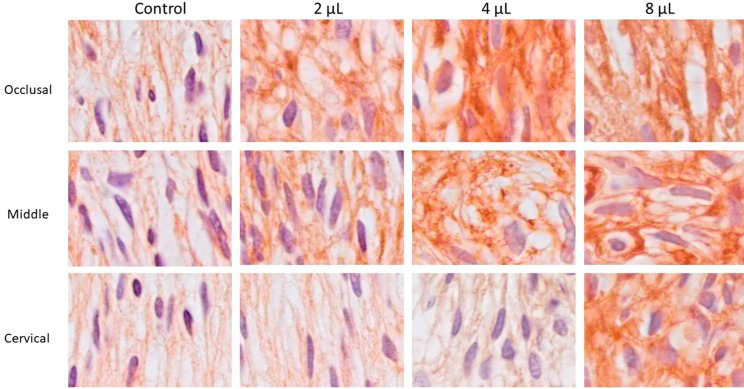
Representative images of the immunohistochemical analysis of nitric oxide synthase, 24 h after bleaching treatment. A low immunolabeling pattern was observed in the occlusal, middle, and cervical thirds in the control group (a; e; i), as well as a moderate immunolabeling pattern in the occlusal and medium thirds in the V2 group (b; f) and in the medium third in the V4 group (g). A severe immunolabeling pattern was observed in all thirds in the V8 group (d; h; l) and in the occlusal third in the V4 group (c). [Immunohistochemistry of nitric oxide synthase: 1,000x]

Figure 3 shows representative images of NOS immunolabeling, and the data are summarized in Table 3. In the occlusal third, the V2 and WG groups showed a similar low immunolabeling pattern (p>0.05), differing significantly from the V4 and V8 groups (p<0.05), which showed severe immunolabeling.

In the middle third, only the WG group showed a low pattern of immunolabeling, differing significantly from the V8 group (p<0.05). V2 and V4 showed moderate immunolabeling, while the V8 group showed severe immunolabeling (p<0.05).

In the cervical third, although the V8 group showed moderate immunolabeling, it did not differ significantly from the other groups (p>0.05).

## Discussion

Although bleaching is considered a dose-dependent therapy,^14,20^ the association between bleaching gel volume and adverse effects on the dental pulp has not yet been explored.^13^ This study aimed to assess the influence of different gel volumes on HP diffusion, pulp inflammation, and oxidative stress in the pulp tissue, and to analyze chromatic changes using different methods. Overall, this study showed that higher bleaching gel volumes increase HP diffusion, pulp inflammation, and oxidative stress, but do not increase bleaching efficacy.

To evaluate bleaching efficacy, we used CIEDE (∆E), CIEDE2000 (∆E_00_), and W_ID_, as the ∆E analysis remains the most common method in clinical research studies on tooth bleaching, and it is interesting to provide comparative and complementary data to the literature.^21,22^ Moreover, the CIEDE2000 analysis shows a better relationship between the hue, saturation, and weights parameters, and differences in lightness, which brings the results closer to the limits of perception of the human eye.^23,24^ Complementary to the ΔE and ΔE_00_ data, the use of W_ID_ shows whether or not the previously measured chromatic change is related to the bleaching effect, offering a more objective analysis.^23,25^

Our findings show that all bleached groups, regardless of the volume applied, had similar ΔE, ΔE_00_, and W_ID_ at the end of treatment. Although we observed the highest values at T4, significant chromatic stabilization occurred in the first bleaching session. Other studies have shown that chromatic changes occur continuously until the third bleaching session.^13^ As this study used bovine teeth without artificial pigmentation, their naturally white hue favored bleaching saturation at the beginning of treatment. However, within the parameters evaluated in this study, bleaching gel volume did not influence chromatic changes or bleaching efficacy, thus corroborating the first null hypothesis. It should be noted that the group that did not receive bleaching gel also showed chromatic changes in the analysis of bleaching efficacy. This change can be explained by the dehydration of the specimens, as they were exposed to the environment for the same periods of time as the groups that received bleaching gel.

Besides the quantitative evaluations performed using a portable spectrophotometer, we also considered the acceptability and perceptibility thresholds proposed by Paravina, et al.^24^ (2017). The authors determined the value of 1.2 as the perceptibility threshold (ability to detect a perceptible difference between two values) and 2.7 as the acceptability threshold (difference considered clinically unacceptable).^24^ Regarding ΔE, all bleached groups showed values within the limits of acceptability and perceptibility at all time points. Regarding the W_ID_ analysis, Perez, et al.^25^ (2017) determined a value of 0.61 as the perceptibility threshold and 2.9 as the acceptability threshold. Thus, at T1, T3, and T4, the V120 group showed a notable but acceptable difference compared with the other groups^25^. It is also worth highlighting that, despite the results of the WG group, when we analyze the W_ID_ values, it is evident that the chromatic changes observed in the samples of the control group did not represent a bleaching effect, but rather the result of the darkening of the sample, suggesting that they result from a process of decomposition of matter (organic substance present in dental tissues).

A previous randomized clinical trial observed that smaller volumes of bleaching gel have a slow but progressive effect compared with treatment with higher volumes in terms of ΔE and ΔE_00_. However, regarding W_ID_, the group that received the largest amount had the greatest bleaching potential.^13^ The continuous bleaching effect obtained may be related to the initial color of the teeth (A2 on the vita scale or darker). This fact corroborates our findings, in which, at the end of treatment, the groups with different volumes were similar to each other. Another randomized clinical trial also observed that bleaching strips with a thin layer of bleaching agent had similar results to the at-home technique, suggesting that small doses of the bleaching product can cause chromatic changes.^26^

In this study, we adopted the methodology proposed by Mottola, Simpson, and Gorin^16^ (1970), improved using APCs,^9^ which allows us to know the concentration of HP diffused into the tooth structure and stabilized by the buffer solution filling the pulp chambers. The presence of HP and other ROS in the pulp cavity has been related to tooth sensitivity, considered the main adverse effects of bleaching.^2,3,4,8^ Our data showed an increase in HP diffusion with increasing gel volume, indicating that the largest gel volume applied (V120) resulted in a diffusion value of 1.78 μg/mL, while the smallest volume showed half the diffusion concentration, thus rejecting the second null hypothesis.

Studies show that after the bleaching gel is applied to the enamel, a chemical imbalance occurs in the area and, in order to restore normality, the peroxide moves to the site of lower concentration, that is, the tooth structure. Our findings suggest that the greater availability of HP in the groups using a larger gel volume intensifies this imbalance, favoring greater HP penetration. Part of the ROS seeks stability by reacting with chromophoric substances and results in the desired bleaching effect. However, the bleaching effect does not respond linearly to the increase in peroxide availability, causing a considerable part of the unreacted ROS to reach the dental pulp.^27^

Balladares, et al.^28^ (2019) suggested that the composition and pH of the bleaching agent could influence the effects of HP on pulp alteration. However, studies such as the one conducted by Canapelle, et al.^29^ (2015) assessed the effect of different bleaching protocols using 35% HP, pH, and gel concentration. They found that replacing or prolonging in-office bleaching gel application did not affect pH, gel concentration, or bleaching response. This is in line with the findings of Marson, et al.^27^ (2015), who studied the degradation rate of 35% HP from several commercial brands and observed that all products significantly reduced the concentration of activated H_2_O_2_ only after 45 minutes. Furthermore, Borges, et al.^30^ (2021) evaluated different commercial brands in terms of bleaching efficacy, enamel microhardness, and roughness of highly concentrated HP gels (ranging from 35% to 40%) using various application protocols. This group concluded that the decomposition of HP during the bleaching session was minimal for all the gels tested. The pH remained stable for the gels and the bleaching protocol (single or multiple application) did not interfere with bleaching efficacy, enamel microhardness, and roughness, using the same gel as in this study. In summary, these studies show that the choice of bleaching gel and application protocol plays a crucial role in achieving effective and safe tooth bleaching, with pH levels remaining stable throughout the process.

Thus, although several studies have evaluated the effects of bleaching therapies on tooth sensitivity, such as antioxidants, photobiomodulation, or even anti-inflammatory drugs,^31,32^ it seems that reducing the gel volume used in the technique can also help prevent symptoms, without harming the aesthetic results. This is because, besides greater HP diffusion, the use of larger bleaching gel volumes also resulted in more intense damage to the pulp, with areas of necrosis and severe inflammatory infiltrate. On the other hand, rats that received the smallest volume had only a moderate inflammatory infiltrate, thus rejecting the third null hypothesis. It should be noted that the values obtained in the *in vitro* analysis of HP permeation cannot be directly correlated with the results of the *in vivo* analysis, due to the differences in the experimental models. However, the increase in inflammation observed when a larger bleaching gel volume was used may suggest a greater amount of HP permeating, both in the *in vitro* and *in vivo* models.

Importantly, the *in vivo* analysis used a shorter application time than that indicated by the manufacturer. This is due to the need to assess the oxidative reactions in the pulp tissue without the occurrence of total coronal pulp necrosis, according to a previous study performed to standardize the rat model^10^. Therefore, considering the significant damage caused to the pulp tissue of bleached teeth^4,8,10^, the commercial bleaching gel with 35% HP diluted to 17.5% HP was chosen for this study. Regarding the volume used in the *in vivo* analysis, 60 µL were required for a gel thickness of 1 mm in the *in vitro* analysis, following the manufacturer’s recommendations. To obtain comparable coverage in rat molars, 4 µL were required. Consequently, the additional groups used half (2 µL) and double (8 µL) this volume, in line with the approach adopted in the *in vitro* analyses.

NOS is a key enzyme in NO synthesis and, consequently, changes in its levels can suggest modulation of NO levels in tissues. In the dental pulp, NO helps maintain homeostasis and participates in the local inflammatory response and tissue repair.^33,34^ In this study, we observed a baseline NOS immunolabeling pattern in the control group, which highlights its importance in maintaining pulp homeostasis. On the other hand, when different bleaching gel volumes were applied, NOS immunolabeling in the dental pulp increased, thus rejecting the final null hypothesis.

The increase in NOS immunolabeling and its presence throughout the different thirds of the dental pulp were shown to be dependent on bleaching gel volume, with the larger volume resulting in greater presence and extent of NOS immunolabeling in the dental pulp. This greater presence of NOS is in line with the greater inflammatory infiltrate, corroborating other studies that point to inflammatory cells as one of the main sources of nitric oxide production.^35^

Esteves, et al.^13^ (2022) also evaluated tooth sensitivity after bleaching with different gel volumes and observed that the groups treated with larger volumes reported greater tooth sensitivity. These findings corroborate that the worsening of the inflammatory condition is directly related to the increase in tooth sensitivity and, consequently, to the amount of ROS that reaches the pulp tissue during treatment. Colares, et al.^36^ (2019) also investigated HP levels after bleaching in fluid samples collected from patients, performing a proteomic study of the samples and a varied analysis of proteins associated with HP, NO synthesis, and neutrophil recruitment. They observed that 21 days after the first bleaching session, an increase in the percentage of proteins related to tissue regeneration remained in the group treated with a lower HP concentration, while an exacerbated inflammatory process remained in the group bleached with 35% HP.^36^

This study has limitations related to the experimental models used, including the discrepancy in the concentration of the bleaching gel used in the *in vitro* and *in vivo* analyses. This is because the *in vivo* model requires a lower bleaching gel concentration to cause less damage to the dental pulp, enabling a better assessment of other variables, such as the different bleaching gel volumes in this case. However, the concentration used in the *in vivo* study proved to be as effective as the higher bleaching gel concentrations used in previous studies.^9,33^ Another limitation is the morphological differences between bovine or rat teeth and human teeth. However, just as this study observed that reducing the amount of bleaching gel can result in a safer treatment with respect to the dental pulp, the previous clinical study showed that a lower bleaching gel volume resulted in reduced tooth sensitivity.^13^

In general, the results obtained in this study suggest that the adverse effects observed in bleaching therapies are also volume-dependent, considering the greater HP diffusion, the higher NOS immunolabeling pattern, and the severe inflammatory infiltrate that occurred when larger volumes were applied. In turn, volume changes were relatively important in the bleaching response, making it prudent to make the products properly, better balancing the aesthetic benefits and biological risks involved in the procedure.

## Conclusion

Bleaching efficacy is not directly linked to the bleaching gel volume applied, but the volume is directly proportional to HP diffusion into the pulp tissue, increasing inflammation and oxidative stress.
